# A 3D LTCC-Based Ceramic Microfluidic System with RF Dielectric Heating of Liquids

**DOI:** 10.3390/ma14237396

**Published:** 2021-12-02

**Authors:** Kostja Makarovič, Darko Belavič, Matjaž Vidmar, Barbara Malič

**Affiliations:** 1 KEKO Equipment, Grajski Trg 15, 8360 Ljubljana, Slovenia; 2 Jožef Stefan Institute, Jamova Cesta 39, 1000 Ljubljana, Slovenia; darko.belavic@ijs.si (D.B.); barbara.malic@ijs.si (B.M.); 3 Centre of Excellence NAMASTE, Jamova Cesta 39, 1000 Ljubljana, Slovenia; 4 Faculty of Electrical Engineering, University of Ljubljana, Tržaška Cesta 25, 1000 Ljubljana, Slovenia; matjaz.vidmar@fe.uni-lj.si

**Keywords:** ceramic microsystem, microfluidics, LTCC, RF dielectric heating

## Abstract

The design, fabrication and functional evaluation of the radio-frequency dielectric heating of liquids in an LTCC-based ceramic microfluidic system are described and discussed. The device, which relies on the dielectric heating of liquids, was fabricated using a low temperature co-fired ceramic (LTCC) technology. A multilayered ceramic structure with integrated electrodes, buried channels and cavities in micro and millimetre scales was fabricated. The structure with the dimensions of 35 mm × 22 mm × 2.4 mm includes a buried cavity with a diameter of 17.3 mm and a volume of 0.3 mL. The top and bottom faces of the cavity consist of silver/palladium electrodes protected with 100 μm thick layers of LTCC. The power, used to heat a polar liquid (water) in the cavity with the volume of 0.3 mL, ranges from 5 to 40 W. This novel application of RF dielectric heating could enable the miniaturization of microfluidic systems in many applications. The working principle of such a device and its efficiency are demonstrated using water as the heated medium.

## 1. Introduction

Radio-frequency (RF) dielectric heating is a well-established heating method that allows for rapid, uniform heating throughout a medium and is widely used in different sectors of industry, such as the wood industry [1], food industry [2], and many others [3,4,5].

The heat is generated within the medium (liquid) and throughout its mass simultaneously due to dipole, atomic, electric, and Maxwell–Wagner effects. The contribution of each mechanism is affected by different frequency ranges, temperature, electric conductivity, moisture content, and the size of polar molecules [6]. Ionic conduction and molecular dipole rotation are dominant mechanisms for RF heating [2].

In the last 30 years, ceramic MEMS and other ceramic microsystems have become popular in many branches, especially in applications where chemical stability, thermal stability, and mechanical stability are important factors [7]. Examples include chemical (micro-)reactors [8], with chemical, fluidic, heating and other functions. Low temperature co-fired ceramic (LTCC) was recognized as a very suitable material for their fabrication [8,9,10,11,12,13,14].

Channels or cavities in a fluidic system are usually heated by resistive heating elements buried between ceramic layers. These heaters are simple and powerful, however, when a high output power is needed, they create a critical gradient of temperature in the ceramic which can cause the microfluidic system to fail [15]. However, the LTCC material can survive relatively high temperature gradients compared to technical ceramics such as Al_2_O_3_ ceramics. The 1 mm × 1 mm × 0.5 mm LTCC block can be heated to more than 300 °C and immediately submerged in water at room temperature without any damage or microcracks [16]. Nevertheless, high gradients and high temperatures in the structure are not beneficial to reliability, connectivity, and fast temperature control.

Alternatively, dielectric heating could be applied in such structures. The cavity should be placed between electrodes and the external high-frequency AC field should be applied to them. Liquids with a high dielectric constant and high dielectric losses are heated throughout the volume of the cavity. The maximal temperature within the structure is defined by the boiling temperature of a heated liquid [1]. 

In this work, we design, fabricate and evaluate the implementation of the RF dielectric heating of water in a ceramic microfluidic structure in LTCC technology. The working principle of this novel approach of high-power heating of liquid is demonstrated in a test microfluidic structure.

## 2. Materials and Methods

**Figure 1:**
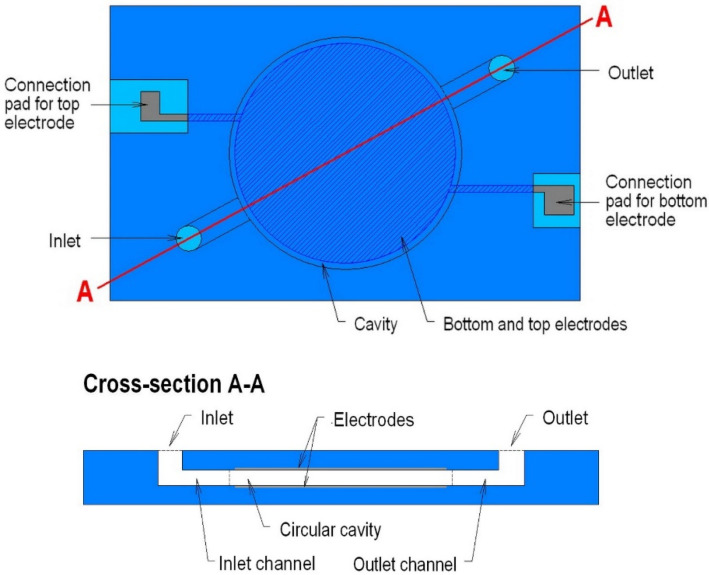
The layout (top view and cross-section) of a 3D LTCC-based ceramic structure for dielectric heating of liquids.

The RF dielectric heating of the liquids was studied in a simple microfluidic system, which was designed as a heating chamber located in the centre of a monolithic ceramic structure with the outer dimensions of 35 mm × 28 mm × 2.4 mm. The layout of the microfluidic system and the cross-sections of the heating chamber are schematically presented in Figure 1. The system contains a heating chamber with a volume of 0.3 mL and diameter of 17.3 mm, integrated bottom and upper electrodes with a diameter of 16.4 mm, electrical interconnections, fluidic channels with the cross-section dimension of 1.9 mm × 1.2 mm and a length of about 5 mm, external electrical contact pads and fluidic ports.

**Figure 2:**
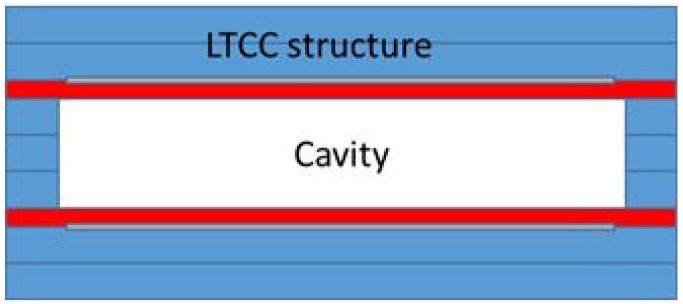
The cross-section (not to scale) of the cavity with the protective LTCC layers, which are presented in red. Note that the thickness of the protective layer is 50 µm or 100 µm. The volume of the cavity remains the same in both cases.

The electrodes are isolated from the fluid by protective LTCC layers of different thicknesses, 50 µm or 100 μm. The structures with such protective LTCC layers are schematically shown in Figure 2.

The three-dimensional (3D) ceramic structure for the microfluidic system with an RF dielectric heater was made from a commercial LTCC tape (KEKO SK-47, KEKO Equipment, Žužemberk, Slovenia) with thickness 254 µm, 100 µm and 50 µm. The properties of this LTCC tape are presented in Ref. [17]. The LTCC tapes were shaped according to the layout by a laser (LM-8UCC, KEKO Equipment, Žužemberk, Slovenia). The internal electrodes and electrically conductive lines and pads were screen-printed with a thick-film silver-palladium paste (KEKO AgPdS-1, KEKO Equipment, Žužemberk, Slovenia) by an automatic screen printer (P-250, KEKO-Equipment, Žužemberk, Slovenia). The punched and patterned layers were collated and laminated for 10 min at a uniaxial pressure of 500 N/cm^2^ and the temperature of 50 °C. This relatively low pressure was used to prevent the deformation of the 3D structure. The LTCC laminate was fired in a single step starting with a heating rate of 7 °C/min up to 450 °C. After a holding time of 1 h to allow a proper binder burnout, the heating proceeded at the heating rate of 10 °C/min up to the sintering temperature of 850 °C for 30 min and cooling with a rate of 10 °C/min. After a visual inspection of the structure, the inlet and outlet ports were bonded with a two-component epoxy glue (Delo, Windach, Germany). The tubes were attached to the inlet and outlet ports. The tightness of the system was tested with an air pressure of about 700 kPa and submerged in water to check for any leakage.

**Figure 3:**
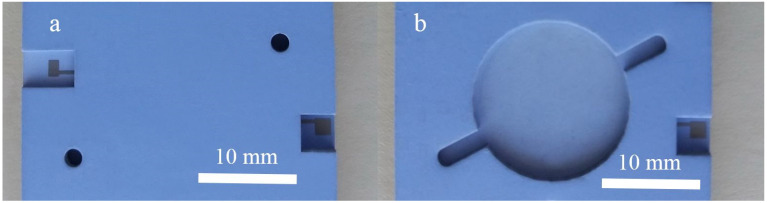
Photo of the structure for dielectric heating of the liquids. A complete structure is marked with (**a**) while the cavity is shown in structure without the lid in (**b**).

A photo of a complete LTCC structure for dielectric heating of the liquids is shown in Figure 3a, and in Figure 3b the structure without the lid is shown. The shape of the heating chamber is clearly seen. 

**Figure 4:**
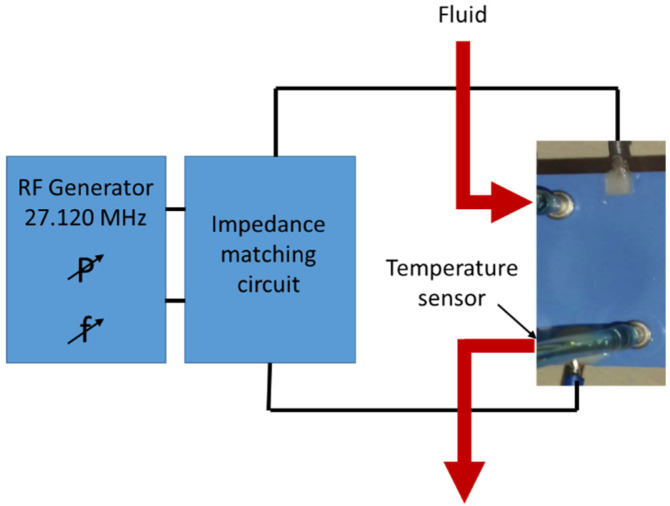
Schematic presentation of the testing setup.

The test setup for dielectric heating of liquids is schematically shown in Figure 4. It consists of a high-frequency power generator (i.e., RF generator). Here, a radio transceiver Yaesu FT-857D (Yaesu, Tokyo, Japan) with selectable output power between 2 and 100 W was used because it enables easy setting and measuring of the output power. The real output power was measured in the range from 5 W to 40 W with an external homemade and calibrated power meter. The dielectric properties of water at different frequencies and temperatures are presented in Table 1 [18]. 

The permittivity is decreasing with increasing frequency and temperature. The dielectric losses are decreasing with increasing temperature and increasing with increasing frequency at a given temperature. The corresponding capacitance contributed to the choice of the frequency. The frequency was set to 27.12 MHz as it is the centre frequency of the ISM band approved by the International Telecommunication Union (ITU). To reduce energy-transfer losses from the transceiver to the electric heater, an impedance-matching electronic circuit was designed and realized, consisting of an adjustable air capacitor and inductance that is changed by using a self-made air coil with a selected number of turns. The impedance matching circuit was then connected to the electrical contact pads at the 3D LTCC structure with copper wires (Alpha Wire, Elizabeth, NJ, USA). 

The fluid with the temperature of about 23 °C entered the dielectric heater through the inlet and the heated fluid exited through the outlet. The flow rate of the fluid and the duration of the flow were controlled. The temperature of the exiting fluid was monitored by a K-type thermocouple (Supco, Allenwood, NJ, USA), which was placed in the outlet tube at a safe distance from the heating chamber to avoid any temperature interference. The temperature was recorded as a function of time while keeping the flow through the chamber constant by using a temperature logger Supco SL500TC (Allenwood, NJ, USA). 

## 3. Results

**Figure 5:**
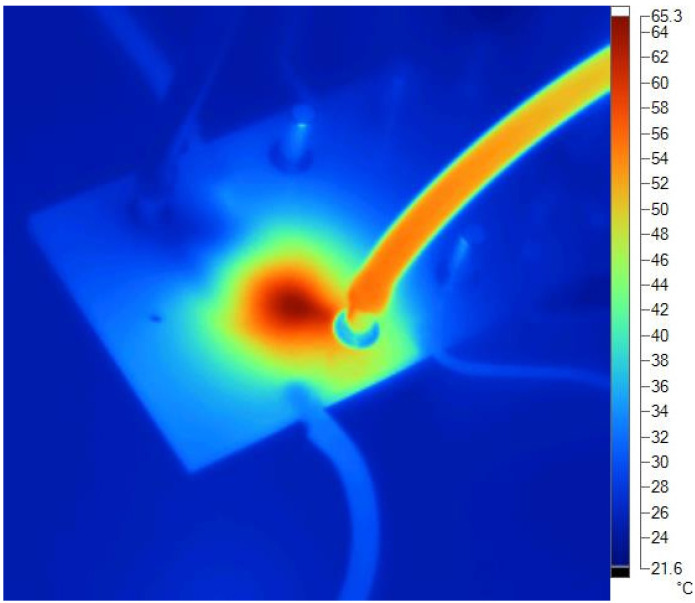
A thermogram of a dielectric heater during the operation at 40 W and flow of 5 mL/min. The colour corresponds to the temperature scale shown on the left side of the image.

The main advantage of a dielectric heater is that it heats only the fluid. A thermogram during the operation at 40 W power with a flow of 5 mL/min is shown in Figure 5. It is clearly seen that the power was concentrated in the centre of the chamber and was not dissipated throughout the structure. This is also a consequence of the low thermal conductivity of LTCC ceramics (about 3 W/(m·K)).

**Figure 6:**
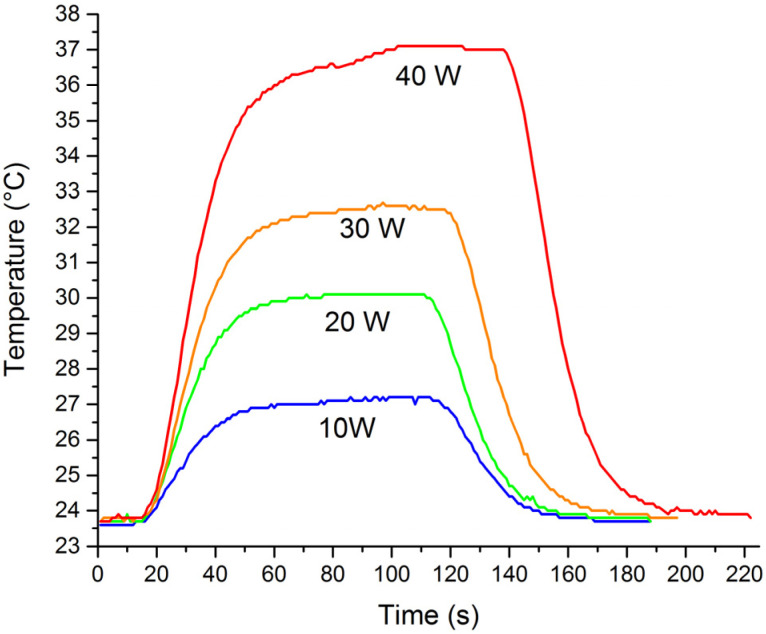
The outlet temperature of water at a constant flow rate for different set heating powers.

In the first experiment, the fluid was water and the RF power was set to 10, 20, 30, and 40 W. At each RF power, the temperature of the exiting fluid at a constant flow rate of 15 mL/min was recorded as a function of time, see Figure 6. Immediately at the onset of heating, the temperature increased until it reached a plateau. A slight increase of the temperature at the plateau at 40 W was ascribed to the local boiling of water, namely, the heating is self-regulating, as the water starts to boil, the capacitance between both cavities drastically decreases and consequently the efficiency of transmitted power drops until the vapour is not present anymore. The set power measured the output power, the difference between the inlet and maximal outlet temperature of the water. The calculated power transferred into water is calculated by $P=q_{m}\cdot c_{p}\cdot \Delta T$, where *P* is power transferred into the water in (W), *q_m_* is mass flow of water (g/s), *c_p_* is heat capacity of water (J/gK), Δ*T* is the difference in temperature between outlet and inlet (K) and the efficiency of power transmitted into water are collected in Table 2. 

The power transmitted into the water increased with increasing output power while the efficiency remained almost constant. At the highest power setting of 40 W, the output power was 33 W. Such power was applied into the cavity with the volume of 0.3 mL which equaled more than 100 W/mL power density applied in such a structure. 

**Figure 7:**
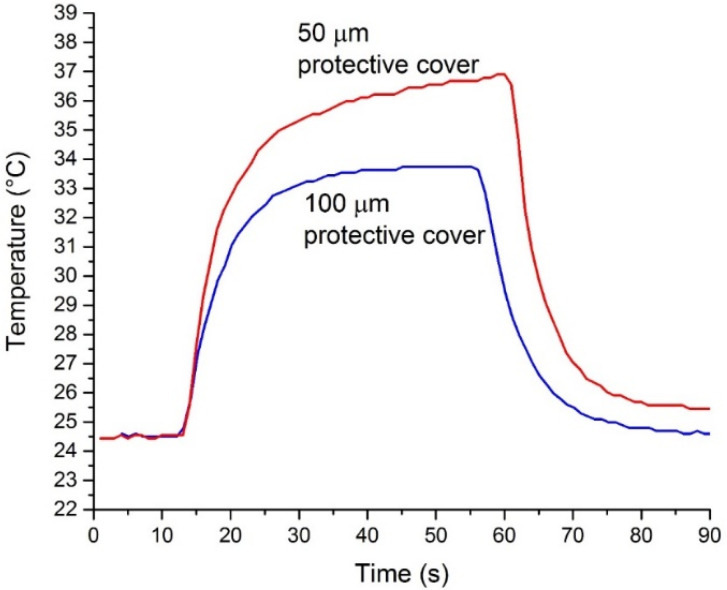
Outlet temperature at the constant water flow and constant power of 20 W for the structures with different thicknesses of protective LTCC layers.

The influence of the thickness of the protective LTCC layer on the power transfer was evaluated. The temperature of the exiting water at the constant water flow at a 20 W set heating power is shown in Figure 7.

The measurements of the calculated power that was transferred into the water and the efficiency of the transmitted power are collected in Table 3.

It is clearly seen that the power transferred into the water was higher in the case of the thinner protective layer as there was a larger fraction of water between the electrodes, please refer to Figure 2. The structure can be electrically represented as two capacitors in series. In the case of a thicker ceramic layer between the electrode and water, the contribution of the ceramic to the impedance and consequently the loss of the power transmitted into the structure is larger.

## 4. Summary and Conclusions

Dielectric heating has been used, according to the authors’ knowledge, for the first time in a ceramic (LTCC) microfluidic structure. The working principle, the use, and its efficiency were demonstrated, using water as the heated medium. The dielectric heater as a part of a ceramic microfluidic 3D structure was realized with LTCC material. When the transmission power was set to 40 W, and the measured transmission power was 33 W, the power density in the structure exceeded 100 W/mL.

The main advantages of dielectric heating of liquids in a LTCC-based ceramic microfluidic system are: (i) it heats only the fluid due to the principle of dielectric heating (ii) it is self-regulated and is determined by the boiling point of the liquid; (iii) the heat dissipation to other parts of the ceramic structure is limited due to the relatively low thermal conductivity of the LTCC material; (iv) dielectric heating is especially convenient for the heating of water, because of its higher dielectric constant; On the other hand, dielectric heating requires an RF generator and an impedance-matching electronic circuit. Here, external devices were used for test purposes, but for a real application, it would be necessary to create a suitable electronic circuit and assemble it into the system.

## Figures and Tables

**Table 1:** The permittivity (**έ**) and dielectric losses (Tan δ) as a function of temperature and frequency.

	0 °C		25 °C		50 °C	
Frequency	έ	Tan δ	έ	Tan δ	έ	Tan δ
0.00	87.90	0.0000	78.36	0.0000	69.88	0.0000
1 kHz	87.90	0.0000	78.36	0.0000	69.88	0.0000
1 MHz	87.90	0.0001	78.36	0.0000	69.88	0.0000
10 MHz	87.90	0.0010	78.36	0.0005	69.88	0.0003
100 MHz	87.89	0.0104	78.36	0.0048	69.88	0.0029
200 MHz	87.86	0.0207	78.35	0.0097	69.88	0.0056
500 MHz	87.65	0.0519	78.31	0.0243	69.87	0.0140

**Table 2:** The set power, measured output power and the difference between the inlet and maximal outlet temperature of the water. The calculated power transferred into water and the efficiency of the power transmitted into the water.

Set Power (W)	Output Power (W)	Δ*T* (K)	Power Transferred into Water (W)	Efficiency of Transmitted Power
40	33	24.7	25.8	0.78
30	22	15.6	16.3	0.74
20	15	10.6	11.1	0.74
10	9	6.3	6.6	0.73

**Table 3:** The power setting, measured output power, difference of the water temperature in inlet and outlet, the calculated heat transferred into the water and the efficiency of the transmitted power depending on the thickness of the protection layer.

Thickness of the Protection Layer (µm)	Output Power (W)	Δ*T* (K)	Power Transferred into Water (W)	Efficiency of Transmitted Power
50	15	12.1	12.7	0.84
100	15	10.6	11.1	0.74

## Data Availability

Not applicable.

## References

[1] Shah Y.T. (2018). Thermal Energy: Sources, Recovery and Applications.

[2] Awuah G.B., Ramaswamy H.S., Tang J. (2014). Radio-Frequency Heating in Food Processing: Principles and Applications.

[3] Brennan J.G., Caballero B. (2003). DRYING|Dielectric and Osmotic Drying. Encyclopedia of Food Sciences and Nutrition.

[4] Fellows P.J., Fellows P.J. (2017). 19-Dielectric, ohmic and infrared heating. Food Processing Technology.

[5] Piyasena P., Dussault C., Koutchma T., Ramaswamy H.S., Awuah G.B. (2003). Radio Frequency Heating of Foods: Principles, Applications and Related Properties—A Review. Crit. Rev. Food Sci. Nutr..

[6] Uan D.G., Cheng M., Wang Y., Tang J. (2004). Dielectric Properties of Mashed Potatoes Relevant to Microwave and Radio-frequency Pasteurization and Sterilization Processes. J. Food Sci..

[7] Birol H., Maeder T., Ryser P. (2005). Low Temperature Co-Fired Ceramic (LTCC) Technology: General Processing Aspects and Fabrication of 3-D Structures for Micro-Fluidic Devices, Sintering 05, 216-219.

[8] Belavic D., Hrovat M., Dolanc G., Zarnik M.S., Holc J., Makarovic K. (2012). Design of LTCC-based Ceramic Structure for Chemical Microreactor. Radioengineering.

[9] Gongora-Rubio M.R., Espinoza-Vallejos P., Sola-Laguna L., Santiago-Avilés J. (2001). Overview of low temperature co-fired ceramics tape technology for meso-system technology (MsST). Sens. Actuators A Phys..

[10] Jiang B., Haber J., Renken A., Muralt P., Kiwi-Minsker L., Maeder T. (2015). Fine structuration of low-temperature co-fired ceramic (LTCC) microreactors. Lab A Chip.

[11] Peterson K.A., Patel K.D., Ho C.K., Rohde S.B., Nordquist C.D., Walker C.A., Wroblewski B.D., Okandan M. (2005). Novel Microsystem Applications with New Techniques in Low-Temperature Co-Fired Ceramics. Int. J. Appl. Ceram. Technol..

[12] Belavič D., Hrovat M., Makarovič K., Dolanč G., Pohar A., Hočevar S., Malič B. (2015). 3D LTCC structure for a large-volume cavity-type chemical microreactor. Microelectron. Int..

[13] Golonka L.J., Malecha K. (2012). LTCC fluidic microsystemsTekočinski LTCC mikrosistemi. Inf. MIDEM.

[14] Malecha K., Golonka L.J., Bałdyga J., Jasińska M., Sobieszuk P. (2009). Serpentine microfluidic mixer made in LTCC. Sens. Actuators B Chem..

[15] Zawada T., Dziedzic A., Golonka L.J. Heat sources for thick-film and LTCC thermal microsystems. Proceedings of the 14th European Microelectronics and Packaging Conference.

[16] Imanaka Y. (2006). Multilayered Low Temperature Cofired Ceramics (LTCC) Technology.

[17] Makarovich K., Meilitsev V., Chigirinsky S. (2018). SK 47 LTCC system by KEKO Equipment Ltd. Electron. Sci. Technol. Bus..

[18] Haynes W.M. (2016). CRC Handbook of Chemistry and Physics.

